# Predictive factors of morbidity after surgical treatment of hepatic hydatid cyst

**Published:** 2012-10-12

**Authors:** Heikal Bedioui, Khouloud Bouslama, Houcine Maghrebi, Jokho Farah, Hichem Ayari, Hamadi Hsairi, Montacer Kacem, Mohamed Jouini, Zoubeir BenSafta

**Affiliations:** 1 Department of Surgery “A” Rabta Hospital, Tunis, Faculty of Medicine of Tunis, Tunisia; 2 Department of Gastroenterology, Charles Nicolle Hospital, Faculty of Medicine of Tunis, Tunisia; 3 Department of Statistics, Ministry of Public Health, Tunisia

**Keywords:** Hydatid cyst, liver, mortality, morbidity, surgery+L11:L15

## Abstract

**Introduction:**

Surgery remains the basic treatment of hepatic hydatid cyst (HHC). However, it is associated with significant morbidity. The aim of our study was to evaluate mortality and morbidity of surgery of the HHC and to highlight the risk factors.

**Methods:**

A retrospective study was conducted from January 1, 1996 to December 31, 2006. 391 patients hospitalized for HHC and operated in the Department of General Surgery “A” of the Rabta Hospital in Tunis, Tunisia.

**Results:**

The overall mortality rate was 0.7% while the overall morbidity rate was 20.4%. About 16.6% suffered from specific complications, while 3.8% suffered from non-specific complications. Predictors of morbidity in a univariate analysis included cysts larger than 9 cm, dome cysts, cysts with bilious contents, type II, III, IV or V on ultrasound classification, fissured cysts and intrabiliary rupture of hepatic hydatid cyst. The multivariate study consisted of independent predictors of disease at the site of the liver dome, the cysto-biliary fistula and intrabiliary rupture of hepatic hydatid cyst

**Conclusion:**

The hepatic hydatid cyst of the dome and the existence of preoperative complications in particular intrabiliary rupture of hepatic hydatid cyst are the main factors of morbidity. A better understanding of these factors allows the surgeon to choose the appropiate surgical technique that is associated with less morbidity.

## Introduction

Hepatic hydatid cyst (HHC), a benign tumor of the liver, is endemic in Tunisia and poses a real public health problem. If not treated, HHC may cause serious complications, preoperative and/or postoperative. The surgeon is often confronted with the choice of the surgical approach to manage HHC cases, radical treatment or conservative treatment. Surgical treatment may lead to significant morbidity. The aim of our study was to evaluate the mortality and morbidity of surgery on hepatic hydatid cysts (HHC) and highlight the risk factors.

## Methods

This is a retrospective study extending over a period of 11 years from 1 January 1996 to December 31, 2006. Patient medical record was reviewed retrospectively. During that period, 465 cases of hepatic hydatid cysts were reviewed, 391 patients were hospitalized and operated in the General Surgery “A” Department of the Rabta Hospital. Recurrent HHC and those associated with intraperitoneal or thoracic localizations were excluded from the study. The characteristics of the hydatid cysts were classified using ultrasound findings (Gharbi classification) [1] and intraoperative findings. We defined postoperative morbidity as the occurrence of a specific or nonspecific complication during the first 30 days of surgery and postoperative mortality by the occurrence of death during the first 30 days postoperatively. Epidemiological, clinical, pathological and therapeutic variables used in the univariate and multivariate analysis were as follows: age, sex, single or multiple cysts, size, content, the site of the cyst, the state of pericyst, ultrasound classification, “fissuring” of the bile ducts, the choice of surgery and the use of omentoplasty or not.

Statistical analysis was performed using SPSS version 16.0. The results were expressed as percentages or averages. The comparison of percentages was made by the chi2 test; Fisher′s exact test for univariate analysis and logistic regression model for multivariate analysis. The significance level was set at 0.05 for all statistical tests.

## Results

Out of the total 391 patients included in this study, 113 were men (28.9%) and 278 were women (71.1%) with a sex ratio of 0.4. The mean age was 41.6 years with the age ranging from 8 to 86 years (Average: 40 ± 16.5). Forty-seven patients (12%) were admitted in emergency for a complicated case of HHC while 344 patients (88%) were admitted to undergo an elective surgery.

Three hundred thirty-three (85%) patients had a single HHC and 58 (15%) cases had multiple cysts (15%). The average size of the cysts was 8.6 cm with a range from 1 to 27 cm. Type IV classification of Gharbi was the most common type (39.6% of cases), followed by type III (28.4%). Types I, II and V were less frequent with 15.9%, 10.5% and 5.6% respectively. The cysts were located in the hepatic dome in 41.5% of cases, the anterior part of right lobe of the liver in 38.5%, left lobe in 18.1%.

Surgical approaches used in resecting the HHC were: 91.3% right subcostal incision and a midline incision in 1% of the cases. Laparoscopic resection was used on only 30 cases (7.7%). Intraoperatively, the content of the cyst was clear in 50% of the cases. In one-fifth of the cases, bilious fluid was detected and in the same proportion, turbid fluid was found. In 12.2% of cases, purulent fluid was discovered in the cysts. The state of pericyst was noticed and mentioned in only 201 cases (43.1%).

The morphology of the cysts (1 = thick, 2 = soft, 3 = unspecified) consisted of 92 (19.7%) thick-walled cysts, 109 (23.4%) soft-walled cysts and 264 (56.9%) unspecified cysts. The number of complicated cysts discovered intraoperatively was 172 (29.6%); infections were 135 (71%), in 4 patients, cyst ruptured in the thorax (2.1%). In two patients (1%), the cyst was found to be compressing the hepatic veins causing a Budd-Chiari syndrome. The 275 (59.1%) uncomplicated cysts that were diagnosed on ultrasound later confirmed intraoperatively.

The number of fissured cyst into the bile ducts was 115 (22.8%). It was a small fistula (1 to 2 mm diameter) in 79 cases (75%) and a large cystobiliary fistula (diameter > 5 mm) in 36 cases (25%).

The conservative treatment was the technique of choice (resection of outside dome surface) in 95% of cases, while 25 patients (5%) were selected to undergo radical treatment. An omentoplastly was used as a means of filling the residual cavity in 49 complicated and non-complicated cysts (10.5%).

The surgical risk was evaluated by the mortality rate which was 0.7% (3 patients). Two female patients, aged respectively 18 and 49 years, died during the intervention in an array of anaphylactic shock by the manipulation of the cyst, even before the injection of scolicide. The third patient was a 16-year old girl admitted to emergency in a state of severe acute cholangitis who died two days post-operation due to severe septic shock.

The overall postoperative morbidity rate was 20.4% (80 patients). Fifteen patients (3.8%) had a nonspecific complication (9 pneumonia, 4 urinary tract infections, decompensated cirrhosis, and a miscarriage in a 1^st^ trimester patient), while 65 patients showed specific complication (16.6%). These specific complications were 35 cases of external biliary fistula (8.9%), infection of the residual cavity in 26 cases (6.6%), 2 cases showed residual membrane in the common bile duct (0.55%) and 2 cases suffered from postoperative hemorrhage (0.55%).

After univariate analysis, the predictors of morbidity are: cyst size of 9 cm or more, cysts locate in the dome, bilious contenting cysts, the type II, III, IV or V ultrasound classification of HHC, fissured cysts into the bile ducts and ruptured cysts into the bile ducts (Table 1). Multivariate analysis showed that cyst localization in the dome of the liver, the cystobiliary fistula and fissuring of cyst into the biliary tract are all independent predictors of morbidity (Table 2).

**Table 1:** Predictive factors of morbidity after surgical treatment of 391 patients hospitalized for hepatic hydatid cyst and operated in the Department of General Surgery “A” of the Rabta Hospital in Tunis, Tunisia from January 1, 1996 to December 31, 2006. (Univariate analysis)

**Type I**	Pure fluid collection - univesicular cyst
**Type II**	Fluid collection with a split wall detached laminated membrane “water lily” sign
**Type III**	Fluid collection with septa daughter cysts
**Type IV**	Heterogenous appearance presence of matrix mimics a solid mass
**Type V**	Reflecting thick walls calcifications

**Table 2:** Predictive factors of morbidity after surgical treatment of 391 patients hospitalized for hepatic hydatid cyst and operated in the Department of General Surgery “A” of the Rabta Hospital in Tunis, Tunisia from January 1, 1996 to December 31, 2006. (Multivariate analysis)

Predictive factors of morbidity	P
**Dome cysts**	< 0.0001
**Size > 9 cm**	< 0.0001
**Type I on ultrasound classification**	0.02
**Bilious contents**	0.002
**Fistulisations in biliary tract**	< 0.0001
**Large fistula**	< 0.0001
Age	0.38
Sex	0.2
Multiple cysts	0.18
Type I, II vs III, IV, V	0.2
Type I, II, III vs IV, V	0.36
Purulent content	0.06
Mastic content	0.4
Type of surgery	0.3
Epiploplasty	0.2

## Discussion

The most frequently encountered morbidities were external biliary fistulas and suppuration of the residual cavity [17, 22, 24, 25]. Intraperitoneal septic complications or persistent bilary fistula postoperative may arise if there is a presence of a residual cavity or cyto-biliary fistula after conservative treatment or if a proper hemostasis or biliostasis is not achieved after radical treatment [2, 17, 26]. Predictors of the disease are not well defined in the literature and the reported results are conflicting and controversial [27]. Our study was limited to the specific predictors of morbidity, particularly postoperative biliary fistula and suppuration of the residual cavity which are the two most common complications especially after conservative treatment. In our study, age and sex did not prove to be predictors of morbidity. This was consistent with several studies but results are conflicting and controversial [8, 24, 27]. However, according to Daradkeh S [3] and El Malki [4], age over 40 years is a predictor of morbidity. This is due to co-morbidites seen in the elderly which increases the overall morbidity.

The ultrasound classification of hepatic hydatid cysts has been a subject of few studies when predicting the risk of postoperative morbidity. According to Demicran [28], type III HHC (using the Gharbi classification [1]) (Table 3) are least likely to complicate postoperatively compared to other types (p = 0.032). On the other hand, Atli [29] stated that Type III cysts were complicated more frequently than the other cysts (p < 0.01). In our study, type II, III, IV and V were more exposed to morbidity than type I. Typically, in type I cysts, the pericyst is often flexible and it is rare to find a large cystobilairy fistula clogged by the membrane. Other studies have not found a correlation between the ultrasound classifications and the occurrence of specific complications [21, 26, 27].

**Table 3:** Gharbi classification of hydatid cysts

Predictive factors of morbidity	P	OR	IC_95%_
Dome cysts	< 0.0001	2.84	1.58 – 5.07
Large fistula	0.02	2.7	1. 13 – 6.46
Fistulisation in biliary tract	0.024	2.3	1.11 – 4.85

Another predictor of postoperative morbidities was the site, particularly cysts located in the dome of the liver. In some studies, the location of cyst being in the dome and the depth of the cyst were found to be a poor prognostic factor for morbidity compared with HHC of left lobe of the liver [4]. In our study, a cyst size greater than 9 cm was found to be a predictor of morbidity. In this sense, the larger the cyst, the greater the risk of the cyst to come into contact with bile ducts and leading to erosion, thus resulting in fissuring of the bile ducts or rupture of the cysts into the bile ducts.

There were no significant differences between post-surgical resection of a single hydatid cyst or mupltiple cysts in the literature. [21, 27, 28]. Our study concluded the same. On the other hand, several authors agreed that cysts filled with bilious or purulent fluid were predictors of morbidity when compared to other contents of the hydatid cysts [27, 28]. This factor was found to be true in our study along with fissuring of the common bile duct. The literature also confirms that the existence of a small fistula or large cystobiliary fistula (diameter> 5 mm) are predictors of morbidity [29–31]. We have not evaluated the medical treatment of hydatid cyst. In fact, in our country (Tunisia), the lack of consensus and the high cost of treatment remains an obstacle to its widespread use, especially since the majority of these patients are from poor families.

Regarding the type of surgery, most studies are unanimous on the fact that the radical method (total cystectomy, lobectomy or hepatectomy) provides better postoperative results than conservative method [7, 15, 21, 30, 32]. In our study, we found no difference in morbidity between the two techniques; however the small number of radical treatments that were used on a selected population cannot permit us to draw a definite conclusion. In fact, the choice of surgical technique is left to the surgeon and is tailored according to the characteristics of the cyst, its site and the experience of the surgeon. The radical treatment appears to a bit extreme for benign disease [30, 33]. Conservative treatment, although simpler and less aggressive, often results in higher morbidity. This method is the preferred treatment in endemic countries like ours where preoperative complications of HHC are more frequent [4, 6, 34]. In addition, several studies show that the omentoplasty significantly decreases morbidity when it fills the cavity of a residual uncomplicated cyst [2]. This theory was not proven in our study probably because this technique was used in a small number of cases including both complicated and uncomplicated cysts.

## Conclusion

The main predictors of morbidity of surgical treatment of hydatid cyst are cysts larger than 9 cm, cysts located in the dome of the liver and cysts ruptured into the bile ducts. Confirmation of these factors in prospective studies would help redefine the surgical strategies used when before a HHC so as to better ensure the best postoperative results.
